# The benefits and challenges of opening toxicology control data

**DOI:** 10.1039/c7tx00071e

**Published:** 2017-07-07

**Authors:** Richard A. Currie, Leigh Dodds

**Affiliations:** a Syngenta Jealott's Hill International Research Centre , Bracknell , Berkshire, RG42 6EY , UK . Email: Richard.currie@syngenta.com; b The Open Data Institute , UK

## Abstract

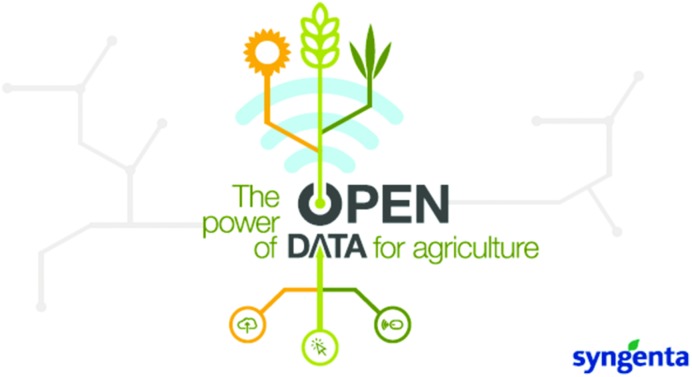
The benefits and challenges of a more open and structured approach to sharing historical control data are highlighted. We invite comment from those who generate, interpret and use toxicology data.

## 

Open data is data that anyone can access, use and share. There is growing evidence that open data can improve the quality of scientific research, enabling researchers to maximise the value gained from collected data.

To reduce unnecessary animal studies, EU and US legislation requires data sharing between organisations seeking to manufacture or import the same substances. But sharing is typically conducted *via* a compensation scheme that restricts other potential uses.

This article discusses the benefits and challenges of a more open and structured approach towards sharing historical control data. This may then serve as a template for the eventual sharing of all toxicology data.

## What trends are impacting data sharing?

Currently sharing of control data happens *via*:

• its provision as supplementary data in academic papers summarising publicly funding studies

• its inclusion in regulatory submissions

• *ad hoc* requests for historical control data, as required to support further analysis and research

Data is usually shared in the form of written reports and there are no data standards for toxicology trial or historical control data.

A changing regulatory environment makes it likely that a more structured approach, based on machine-readable data will become the norm. By December 2016, the FDA mandate that data from toxicology studies supporting the submission of new drugs must be made available in the SEND†
†https://www.cdisc.org/standards/foundational/send. format.

The EPA and other regulatory bodies are likely to adopt similar criteria for the registration of chemicals.

## Why publish historical control data as open data?

Open publication of control data might be beneficial for several reasons:

• **Enabling new and innovative uses** – Aggregating data could provide additional insights into the biology of the test species. Models used in computational toxicology will also benefit from access to additional datasets.

• **Improving scientific practice** – The Registry of Industrial Toxicology Animal-data (RITA)‡
‡http://reni.item.fraunhofer.de/reni/public/rita/. project has demonstrated the benefits of gathering and harmonising data to improve the practice of pathology. These successes could extend into other areas of diagnostic or research practice.

• **Streamlining regulatory processes** – Streamlining the regulatory process has benefits for both the regulators and the registrants. Machine-readable data will enable more automated analysis of submissions, while easy access to historical control data will simplify requests for additional evidence and re-registration of existing substances. A data-driven approach towards regulation may also increase public confidence in the process.

There is also the ethical argument that researchers should be trying to maximise the value gained from animal testing data. Historical control data contains no sensitive or proprietary information and should be uncontroversial to publish more widely.

## What are the potential challenges with publishing open control data?

There are a number of potential challenges that will face successful publication and reuse of open control data:

• **Creating standard data formats** – Existing technical specifications, *e.g.* SEND, may need to be extended, or new standards may need to be developed.

• **Data harmonization** – For control data to be comparable it must have both clear provenance and conform to standardised nomenclature and taxonomies that enable reuse.

• **Costs of publication and distribution** – Beyond the growing number of public repositories for open scientific data additional data infrastructure, tools and processes may be required.

The legislative requirements to share trial data are currently fulfilled *via* custom licence agreements and typically involves some level of compensation. Contract Research Organisations (CROs) and those organisations funding toxicology studies are also currently reimbursed for their data collection costs.

While CROs currently share data with their customers they may be wary about sharing data more widely, particularly if it is currently viewed as a revenue-generating asset.

These factors may create resistance towards the adoption of more open approaches.

## Next steps

While there are several challenges to be faced, the authors believe that the potential benefits make this idea worth further exploration. We would like to invite comments from the community to capture their views on the potential utility and feasibility of sharing toxicology control data and their willingness to participate in a workshop to explore this topic further. In particular we encourage academic, commercial, regulatory, governmental and non-governmental organisations and individuals that generate, define and use toxicology control data; those who have experience of defining data formats and public standards and creating public resources/repositories of such data; and those with an interest in the 3Rs to share their views. To that end we have created a web-based survey to capture views and serve as a way to gauge interest in a potential future workshop, see:

http://bit.ly/2udZG7v.

## Conflict of interest

There are no conflicts of interest to declare.

